# Controlling bi-anisotropy in infrared metamaterials using three-dimensional split-ring-resonators for purely magnetic resonance

**DOI:** 10.1038/s41598-017-07026-w

**Published:** 2017-07-27

**Authors:** Yuto Moritake, Takuo Tanaka

**Affiliations:** 1 Innovative Photon Manipulation Research Team, RIKEN Center for Advanced Photonics, Wako, 351-0198 Saitama Japan; 2 Metamaterial Laboratory, RIKEN, Wako, 351-0198 Saitama Japan; 3 School of Materials and Chemical Technology, Tokyo Institute of Technology, Tokyo, 152-8550 Japan

## Abstract

We propose and demonstrate the strategy to control bi-anisotropic response in three-dimensional split-ring-resonators (3D-SRRs) array for purely magnetic resonance in the mid-infrared region. By using a metal-stress-driven self-folding method, inversion symmetry along a propagation axis of 3D-SRRs was controlled. The inversion symmetry of 3D-SRRs realized non-bi-anisotropic response of a magnetic resonant mode at around 10 μm in wavelength resulting in purely magnetic resonance with high transmission of 70%. Highly transparent purely magnetic artificial elements demonstrated in this study will be a key component for functional applications using artificial magnetism at the optical frequencies.

## Introduction

Naturally occurring materials have negligible magnetism at optical frequencies because interaction between a magnetic component of light and matters are significantly weak compared with the interaction with electric components of light, meaning that permeability of materials is always unity in the optical frequencies. This situation was fundamentally changed by emergence of metamaterials which can realize artificial magnetism at optical frequencies^1^. Metamaterials are artificial materials composed of sub-wavelength structures and they allow us to design their electromagnetic properties even if these properties cannot be found in nature. Artificial magnetism in metamaterials has opened new opportunities to access intriguing phenomena including negative refractive index^1–3^ and s-polarization Brewster effects^4, 5^. Especially, negative refractive index and relating phenomena have attracted significant attention from researchers due to exciting applications including perfect lens, cloaking devices, and so on^6–8^.

Artificial magnetism in metamaterials was firstly demonstrated in GHz region by using split ring resonators (SRRs) consisting of inductive metal rings with gaps^2^. Magnetic component of light can couple to eigen modes with circulating current inducing magnetic dipole and effective permeability at the magnetic resonance in SRRs becomes different from unity. Since magnetic response was demonstrated using SRRs, numerous studies on artificial magnetism using SRRs have been reported so far^1–5, 7, 9^. Miniaturization of the SRRs brings the resonant frequency of artificial magnetism to the optical region^1, 9^.

To demonstrate artificial magnetism in the optical region using SRRs experimentally, planar type SRRs that were fabricated on the surface of the substrate are generally used due to restriction of sample fabrication. Since the excited magnetic dipole in planar SRRs is normal to a substrate, magnetic components of normal incident light cannot directly couple to the magnetic resonant modes while only electric components of light can couple to magnetic resonant modes^10^. Therefore, planar SRRs cannot act as “purely magnetic elements” in which magnetic dipoles are excited only by the magnetic component of light. In order to create magnetic response for normal incident light, three-dimensional (3D) SRRs that are standing on the substrate are necessary. So far, several kinds of 3D-SRRs are demonstrated in THz and optical region^11–17^ for direct coupling between magnetic components of light and magnetic dipoles in SRRs. However, even in the case of 3D-SRRs, magnetic dipoles excited by electric component of light are created due to bi-anisotropic response if structures have no inversion symmetry along propagation direction. It means that at bi-anisotropic response in metamaterials, electric (magnetic) response can be excited by not only electric (magnetic) component but also magnetic (electric) component of light, which results in unwanted complex electro-magnetic coupling at the resonance of SRRs^9, 18^.

**Figure 1:**
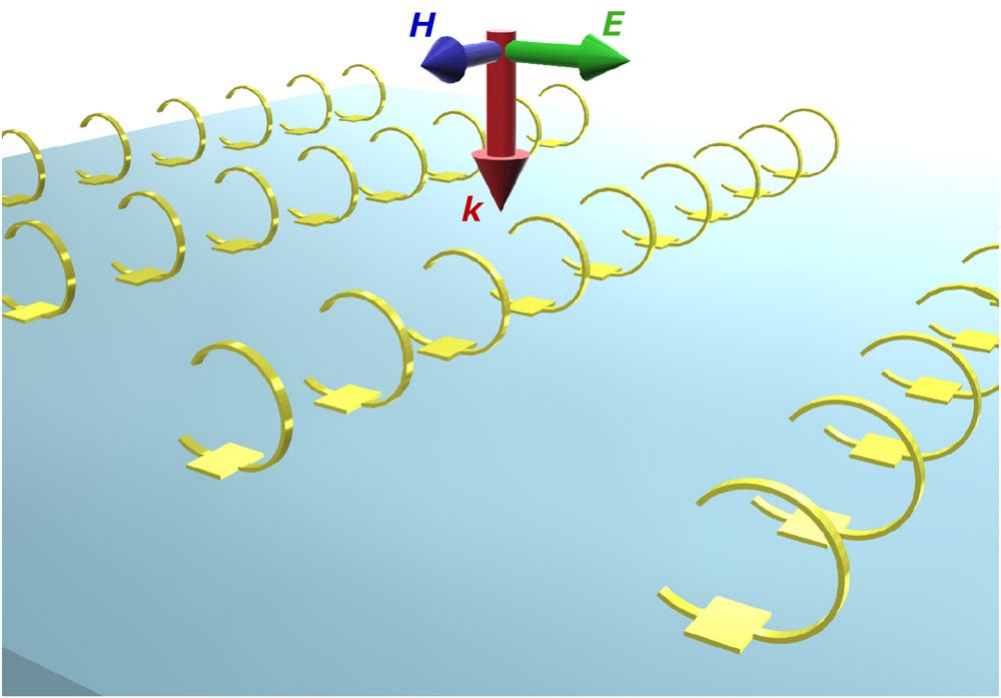
A schematic of 3D-SRRs with inversion symmetry along *z-*axis fabricated by using a metal-stress-driven self-folding method for non-bi-anisotropic response.

To eliminate bi-anisotropic response of metamaterial structures for purely magnetic elements, not only 3D structures but also inversion symmetry along propagation direction of an incident light (*z-*axis) are necessary^11^. For single-cut-SRRs, inversion symmetry along *z-*axis can be manipulated by controlling the gap orientation. When the gap is positioned at the side of the 3D-SRRs, the structures become symmetry along *z-*axis and bi-anisotropic response should be eliminated resulting in magnetic resonance only (Fig. 1). In this study, we propose and demonstrate the strategy to control bi-anisotropy of 3D-SRRs for purely magnetic resonance in the mid-infrared region. 3D-SRRs with inversion symmetry along *z-*axis are fabricated by using a metal-stress-driven self-folding method^15–17^. Non-bi-anisotropic response in 3D-SRRs realizes highly transparent purely magnetic resonance, which is confirmed by effective parameter retrieving.

## Results and Discussion

**Figure 2:**
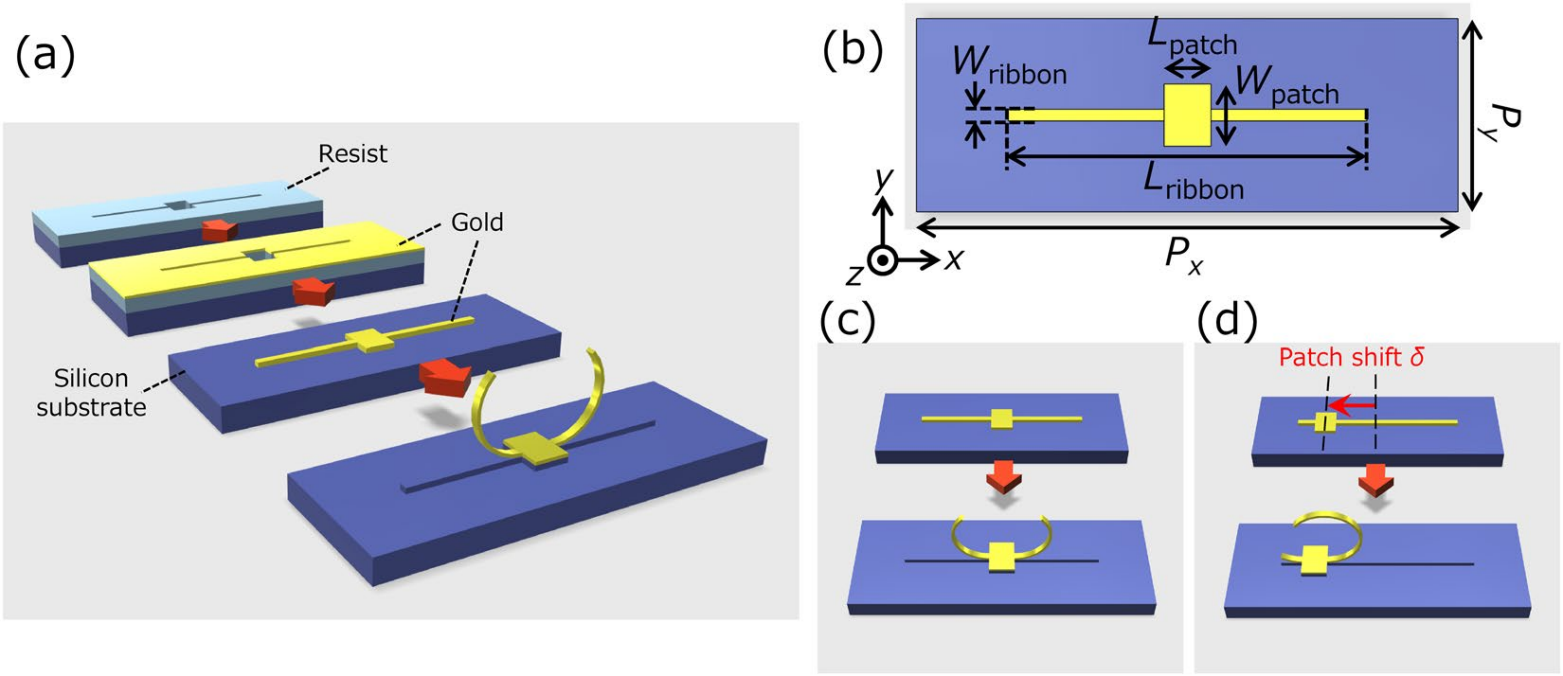
(**a**) Fabrication step of a metal-stress-driven self-folding method. (**b**) The design of 2D gold patterns. (**c**) 3D structures after folding with and without the patch shift *δ*.

Fabrication of 3D-SRRs was performed by using a metal stress-driven self-folding method we have developed so far as shown in Fig. 2a ^19–21^. Firstly, two-dimensional (2D) gold patterns are formed on a Si substrate by using conventional lift-off method. In this method, patterning on a resist and evaporation of gold are performed by using electron beam lithography and thermal evaporator, respectively. The design of 2D gold patterns is summarized in Fig. 2b. The 2D patterns are composed of a ribbon (length *L*_ribbon_ = 4.60 μm, width *W*_ribbon_ = 0.15 μm) and a patch (length *L*_patch_ = 0.60 μm, width *W*_patch_ = 0.80 μm). The periodic lattice constants along *x-* (*P*_*x*_) and *y*-axis (*P*_*x*_) are 7.00 and 2.50 μm, respectively. The thickness of gold patterns is 60 nm. The Si substrate with 2D patterns is isotropically etched using inductive coupled plasma reactive ion etching (ICP-RIE, SAMCO, RIE200iPT). When Si under the gold ribbons is completely etched and the ribbons are released from the surface of the Si substrate, intrinsic stress in deposited metal film spontaneously induces folding of the ribbons. Here, intrinsic stress in the gold film is originated from the lattice mismatch, grain boundaries, difference of thermal expansion coefficient, impurities in the gold film, and deposition method^19^. For example, in thermal evaporation we used, temperature of the metal and substrate is different during deposition if substrates are not heated. Therefore, shrinking force occurs in the metal film after the sample was cooled to room temperature, resulting in intrinsic stress. The role of the center patch is an anchor for the ribbons not to be peeled off from the substrate after release. Owing to self-organized nature of the process, this method allows mass and easy fabrication of 3D structures.

To control inversion symmetry, namely, bi-anisotropy of the 3D-SRRs, the position of the anchor patch was shifted along the ribbons. When the patch is located at the center of the ribbons, the gap is also formed at the center of the 3D rings as shown in Fig. 2c. If the patch shift *δ* is introduced, the 2D gold patterns composed of the ribbons and patches becomes asymmetrical and the gap position rotates after ring formation as shown in Fig. 2d. The patch shift *δ* is varied from 0.0 to 1.5 μm. Shifting the patches realizes continuous control of the gap position in 3D-SRR. This continuous variation of the gap position is one of the advantages of our fabrication method realizing circular 3D-SRRs while most of 3D-SRRs in preceding works have rectangular shapes^11–17^. In the case of *δ* = 1.5 μm, the gaps are nearly positioned at the side of 3D-SRRs and 3D-SRRs have inversion symmetry along *z-*axis.

**Figure 3:**
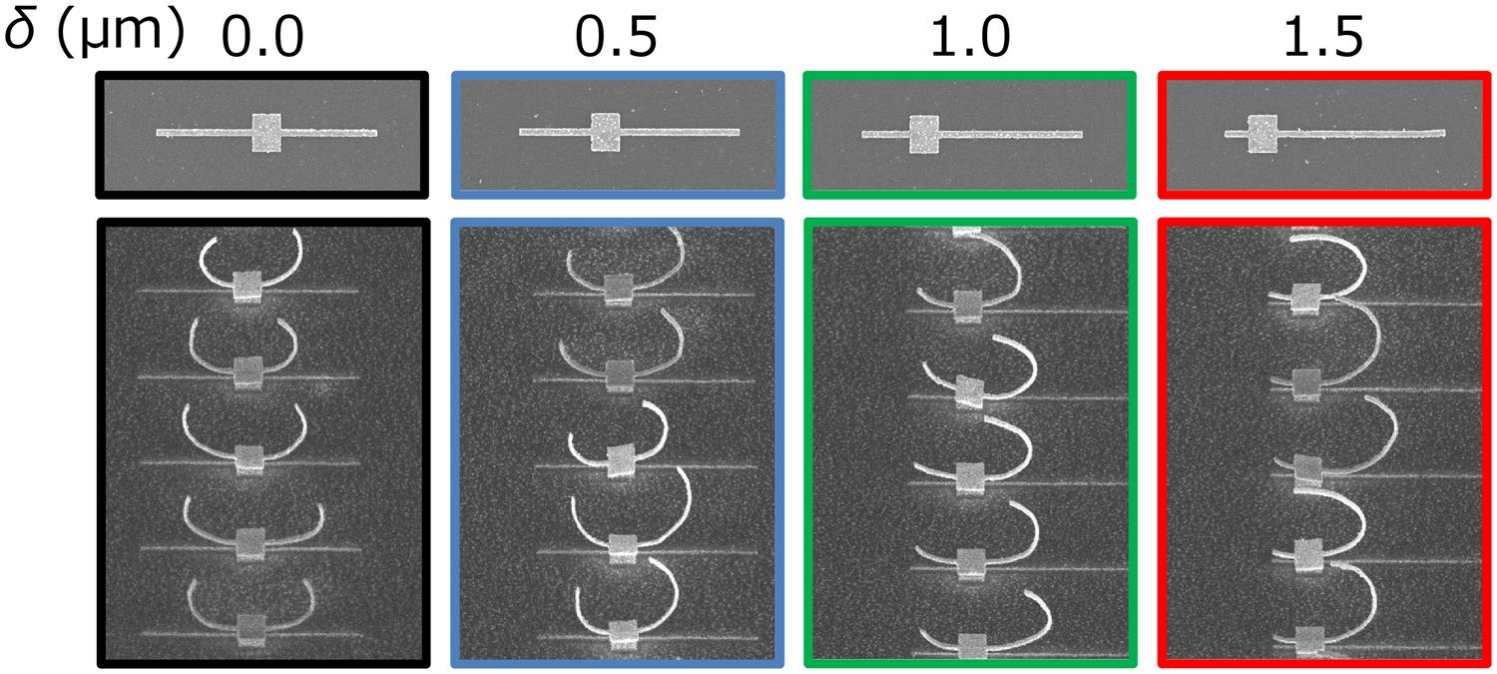
SEM images of 2D gold patterns and 3D-SRRs for respective *δ*.

Figure 3 shows scanning electron microscope (SEM) images of the fabricated 2D gold patterns for respective *δ* and corresponding 3D structures after folding. The gap positions were successfully controlled by the patch shift and rotating the gap positions improves inversion symmetry along *z-*axis.

**Figure 4:**
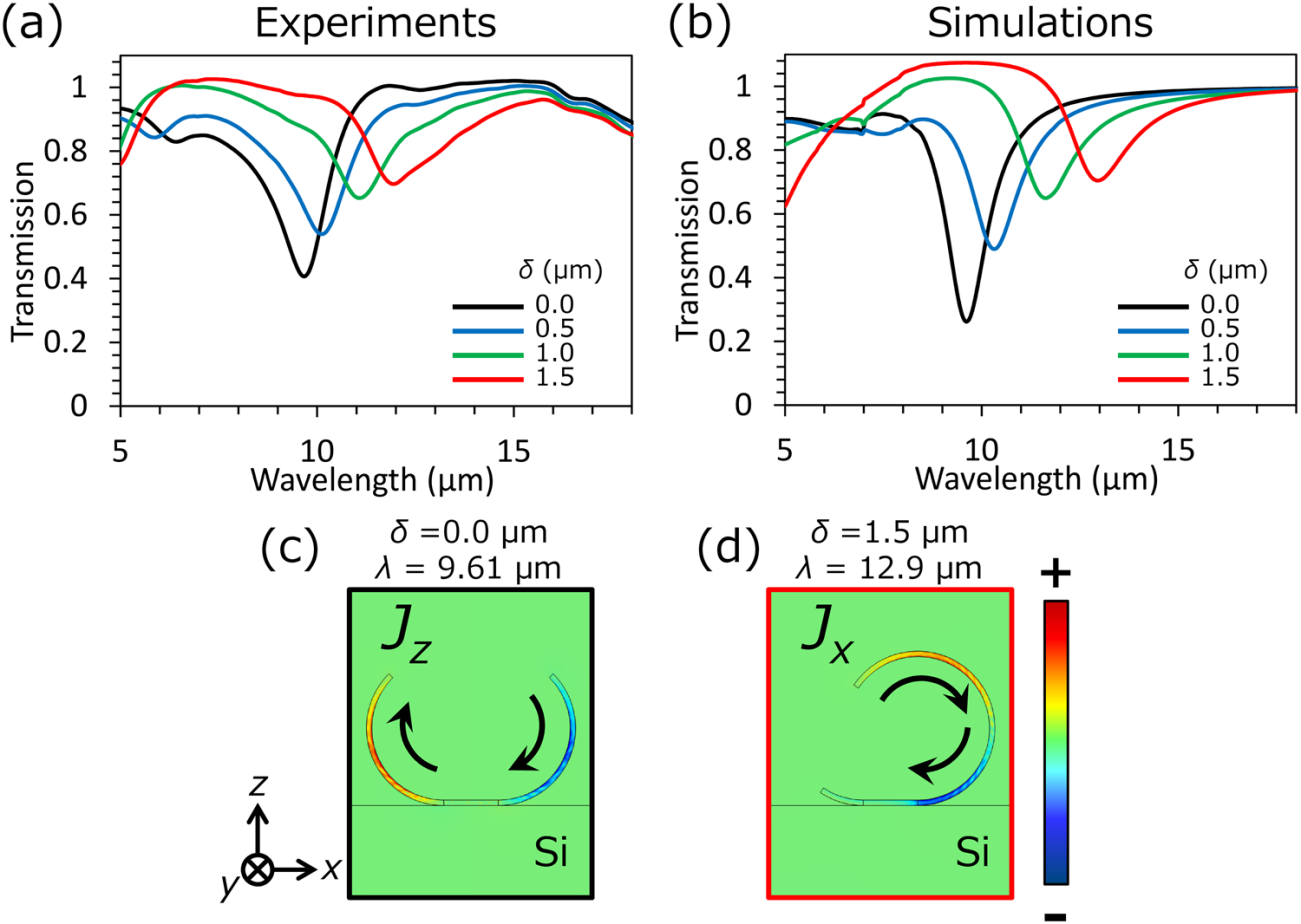
(**a**) Experimental and (**b**) simulated transmission spectra for 3D-SRRs with the patch shift *δ*. (**c**) A *z-*component of a current distribution flowing along the ring in the case of *δ* = 0.0 μm. (**d**) An *x-*component of a current distribution flowing along the ring in the case of *δ* = 1.5 μm.

Optical characterization was performed by microscopic Fourier-transform infrared (FT-IR) measurements, and the results of transmission spectra are shown in Fig. 4a. Incidence was normal to a substrate and its polarization direction was along to the ribbons of 3D-SRRs (*x-*axis). To eliminate the absorptive effect of the substrate, each spectrum is normalized by that of the bare Si substrate. Magnetic resonances are observed at wavelengths from 10 to 12 μm, and they red-shifts as *δ* increases. Numerical simulation using a finite-element method software (COMSOL) was also carried out. The results are shown in Fig. 4b, and these agree well with the experiments. Simulated current distributions at transmission dips in Fig. 4b are shown in Fig. 4c,d. Figure 4c,d corresponds to *z-* (*x-*) components of current flowing along the 3D-SRRs with *δ* = 0.0 (1.5) μm. Circulating currents along the rings is clearly observed, which means the magnetic resonant mode is excited in both cases. At the magnetic resonance, electric fields concentrate at the both ends of the ring due to the charge accumulation. When *δ* increases and 3D-SRRs rotate in *x-z* plane, the one end of the ring approaches the Si substrate with refractive index of 3.4, which leads to the red shift of the magnetic resonance as observed in Fig. 4a.

**Figure 5:**
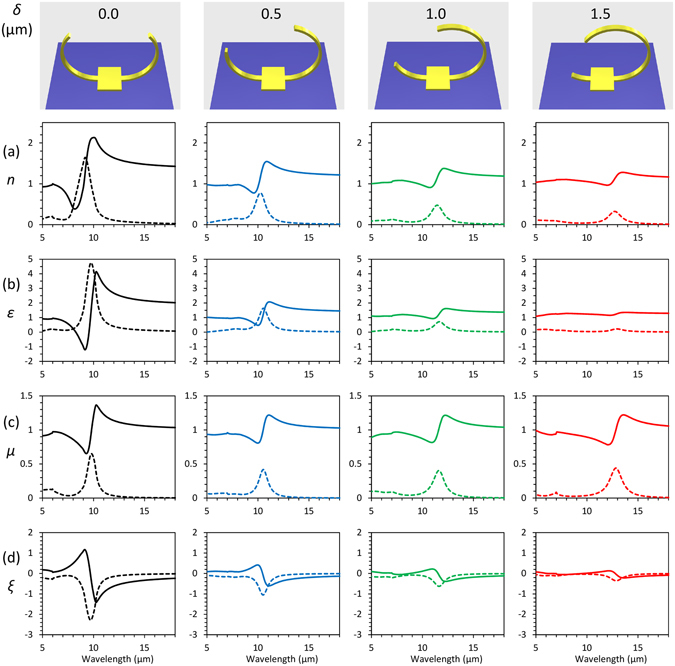
Retrieved effective parameters for 3D-SRRs on a Si substrate for respective *δ*. (**a**) Refractive index (*n*), (**b**) relative permittivity (*ε)*, (**c**) relative permeability (*μ)*, and (**d**) bi-anisotropic parameter (*ξ)* for different *δ*. Solid and dashed lines correspond to real and imaginary parts of effective parameters, respectively.

In order to confirm controllability of bi-anisotropic response in 3D-SRRs by improving inversion symmetry, effective permittivity, permeability, and bi-anisotropic parameters of the metamaterials were retrieved by following ref. 22. The relation between electromagnetic fields in bi-anisotropic materials can be expressed as

1 $$(\begin{array}{c}D\\ B\end{array})=(\begin{array}{cc}{\varepsilon }_{0}\varepsilon  & -i\xi /{c}_{0}\\ i\xi /{c}_{0} & {\mu }_{0}\mu \end{array})(\begin{array}{c}E\\ H\end{array})$$

where *ε*_0_, *μ*_0_, *ε*, and *μ* are vacuum permittivity, vacuum permeability, relative permittivity, and relative permeability, respectively. *c*_0_ is the speed of light in a vacuum, and *ξ* is a bi-anisotropy parameter, which describes the bi-anisotropic response. The effective parameters can be retrieved using complex transmission and reflection coefficients calculated by COMSOL. The retrieved effective parameters are summarized in Fig. 5. In the case of *δ* = 0.0, all effective parameters show Lorentzian type dispersive responses at the magnetic resonant wavelength as shown in Fig. 5. From Fig. 5b,c and d, increase in *δ* leads the reduction of the electric and bi-anisotropic responses while magnetic response remains almost unchanged, meaning the magnetic resonance at around 10 μm is excited by only magnetic component of light by improving inversion symmetry along *z-*axis. At magnetic resonance in SRRs, not only magnetic dipole but also electric one are induced. Since the induced electric dipoles are parallel to the gap direction of SRRs, rotating the gap makes electric dipoles normal to the surface of the substrate. Therefore, an electric component of incident light cannot couple with the magnetic resonant mode through the electric dipole excitation, and it eliminates alteration in permittivity and bi-anisotropic parameters. Reduction of electric coupling leads to low reflection at the resonance and the transmission becomes high reaching to around 70%.

Finally, we would like to point out the effect of a substrate on bi-anisotropic response. Presence of a substrate induces bi-anisotropy of optical metamaterials because of large permittivity difference between air and a substrate inherently breaks the inversion symmetry along *z-*axis. Therefore, even in 3D structures with inversion symmetry along *z-*axis, bi-anisotropic response remains^11^. However, as shown in Fig. 5d, our 3D-SRR with inversion symmetry along *z-*axis (*δ* = 1.5 μm) has negligible bi-anisotropic response, although the structures are standing on Si substrate, which has large refractive index of 3.4. This is because our 3D ring structure is attaching to the substrate with a small portion of its ring structure compared with the rectangular SRRs. This is another advantage of our self-folding method using a releasing process. Remaining small bi-anisotropic response is because of imperfectness of the inversion symmetry due to the anchor patch.

## Conclusions

In summary, we proposed and demonstrated the strategy to control bi-anisotropic response in 3D-SRRs for purely magnetic resonance in the mid-infrared region. 3D-SRRs were fabricated by the metal-stress-driven self-folding method. By introducing the patch shift into the self-folding method, the gap orientation of 3D-SRRs were freely controlled. When the gap is positioned at the side of 3D-SRR, inversion symmetry along *z-*axis were achieved and bi-anisotropic response of magnetic resonance at around 10 μm was eliminated. Owing to this elimination of the bi-anisotropic response, magnetic dipoles at the resonance was excited by only magnetic component of light, and magnetic resonance became purely magnetic. The elimination of bi-anisotropic response also decreases electric response resulting in low reflectance and high transmission of 70%. Despite presence of the Si substrate, bi-anisotropic response was almost eliminated in proposed 3D-SRRs, which may be due to small portion of attaching parts between the structures and the substrate. Our fabrication method takes great advantages for realizing purely magnetic elements in metamaterials because of controllability of inversion symmetry and negligible effect of substrate on bi-anisotropy. Highly transparent purely magnetic artificial elements demonstrated in this study will be a key component for functional applications using artificial magnetism at the optical frequencies.
